# Obstructive Sleep Apnea Risk and Stroke among Blacks with Metabolic Syndrome: Results from Metabolic Syndrome Outcome (MetSO) Registry

**DOI:** 10.15344/2456-8007/2020/143

**Published:** 2020-02-26

**Authors:** April J. Rogers, Ian Kaplan, Alicia Chung, Samy I. McFarlane, Girardin Jean-Louis

**Affiliations:** 1 St. John’s University, Division of Health and Human Services, College of Professional Studies, Queens, New York, USA; 2 SUNY Down State Medical, Department of Internal Medicine Center Brooklyn, New York, USA; 3 Center for Healthful Behavior Change (CHBC), Department of Population Health, NYU Grossman School of Medicine, New York, NY, USA

**Keywords:** MetSO, Blacks, Metabolic Syndrome, Obesity, Obstructive Sleep Apnea, Risk factor, Stroke

## Abstract

**Introduction:**

The American Stroke Association estimates that stroke is the fifth leading cause of death in the United States. According to the Center for Disease Control and Prevention someone in the United States has a stoke every 40 seconds, affecting more than 795,000 people of which 140,000 result in death [1]. Emerging evidence suggests that obstructive sleep apnea (OSA) is a strong risk factor for stroke. This study using The Metabolic Syndrome Outcome (MetSO) registry explored whether blacks at risk for obstructive sleep apnea (OSA) are at greater risk for a stroke.

**Method:**

The present study utilized data from the MetSO study, an NIH-funded cohort study of blacks with metabolic syndrome (MetS). Patients were diagnosed with MetS using standard criteria articulated in the joint interim statement for harmonizing the MetS. The study assessed OSA risk using the Apnea Risk Evaluation System (ARES); defining high risk as a total ARES score ≥6. Data was coded and analyzed by an experienced statistician using SPSS 20.0.

**Results:**

A total of 1035 participants were screened for MetS in the MetSO registry. During the data collection period 875 participants were enrolled during the time of analysis. The average age of the sample was 62±14 years (range: 20–97); 71% were female, and all were of black race/ethnicity. Seventy-one percent reported finishing high school, and 43% reported annual income <10K. Descriptive analyses showed 93% of the participants were diagnosed with hypertension; 61%, diabetes; 72%, dyslipidemia; 90% were overweight/obese; 33% had a history of heart disease and 10% had a stroke history. Using the ARES screener, we estimated that 48% were at high risk for OSA. Logistic regression analysis, adjusting for age and gender, showed that patients at high risk for OSA had a nearly three-fold increase in the odds of having a stroke (OR = 2.79, 95% CI: 1.64–4.73).

**Conclusion:**

In the MetSO registry, a cohort of blacks with MetS, the prevalence of stroke is greater than in the general US population. Blacks at risk for OSA are particularly vulnerable to experiencing a stroke.

## Introduction

In the United States, stroke is one of the top 5 leading cause of death, in addition to being a leading cause of adult disability [2–4]. An estimated 140,000 people die annually in the US as a direct result of a stroke, leaving roughly 795,000 to suffer the consequences of disabilities, giving rise to considerable economic loss and social issues [1,2,4,5]. Research shows that 63% of patients who experience an acute stroke or transient ischemic attack manifest obstructive sleep apnea (OSA) having an Apnea-Hypopnea index (AHI) score greater than 10 [6]. Obstructive sleep apnea (OSA), a condition in which patients experience recurrent apnea and hypopnea episodes due to complete or partial collapse of the upper airway [7]. The repeated cessations of breathing (apneas) and slow or shallow breathing (hypopneas) during sleep that characterize OSA are accompanied by sleep disturbances, hemodynamic changes and heighten thrombotic potential. Mechanisms of pathology in OSA that are important to consider include sympathetic activation, intra-thoracic pressure changes and oxidative stress. Additionally, endothelial damage with subsequent platelet activation and increased systemic inflammation potentiating vasoconstriction are implicated in the atherosclerotic process and are important factors to consider in disease progression. Hypoxemia appears to drive these pathways [8]. It is for these reasons that OSA is a strong risk factor for hypertension (including resistant hypertension), coronary artery disease, renal disease, heart failure, arrhythmias and stroke [3,4,7,9–11].

An important particularly vulnerable sub-group within black populations with OSA are black patients with metabolic syndrome. Metabolic Syndrome (MetS) is a constellation of clinical and metabolic risk factors that include abdominal obesity, dyslipidemia, glucose intolerance, and hypertension. In general, blacks have lower prevalence of MetS when compared to whites, but suffer disproportionately from higher cardiovascular mortality and type two diabetes mellitus [12]. It is unclear whether OSA and metabolic syndrome work synergistically within the black population to place this vulnerable sub-group at an even greater risk of stroke. Studies have speculated, that OSA may create and/or intensify the pre-existing negative components of metabolic syndrome (MetS), suggesting OSA to play a vital role in the pathogenesis of metabolic dysfunction [13]. There is a surprising lack of research regarding the risk of OSA and stroke among blacks with MetS. Given the increased risk of morbidity and mortality associated with OSA, and the undeniable economic burden this places on the US healthcare system, this study sought to investigate blacks with MetS and their risk for OSA and stroke within the Metabolic Syndrome Outcome (MetSO) trial.

## Method

Data were collected as part of the Metabolic Syndrome Outcome study (MetSO), an NHLBI funded registry of 1035 blacks with metabolic syndrome in a primary-care setting in four clinics associated with SUNY Downstate Medical Center in Brooklyn, NY. During the data collection period with 875 patients were enrolled during the time of analysis. Validated questionnaires were administered in order to obtain data on sleep apnea risk, day time sleepiness, demographic, anthropometric data, and diseases associated with risk for sleep apnea (i.e. hypertension, diabetes, heart disease, and stroke) (Allscripts, Sunrise Enterprise).

Patients were diagnosed with metabolic syndrome using the National Heart, Lung and Blood Institute and the American Heart Association guidelines. According to these guidelines, metabolic syndrome is diagnosed when a patient has at least three of the following five conditions: 1) fasting glucose ≥ 100 mg/dl or receiving treatment for hyperglycemia, 2) blood pressure ≥ 130/ 85 mmHg or receiving drug therapy for hypertension, 3) triglycerides ≥ 150 mg/dl or receiving drug treatment for hypertriglyceridemia, 4) HDL-C<40mg/dl in men or <50 mg/dl in women or receiving drug therapy for HDL-C and 5) a waist circumference ≥ 102 cm (40 in) in men or ≥ 88 cm (35 in) in women [14]. Physician-diagnosed conditions were obtained using an electronic medical record (EMR) system (Allscript, Sunrise Enterprise). We used the Apnea Risk Evaluation System (ARES) to identify individuals who were at OSA risk because of its accuracy in evaluating populations with a large pretest OSA probability. Data solicited included socio-demographics, diseases associated with OSA, the Epworth Sleepiness Scale (ESS), and frequency of breathing abnormalities. The ESS questionnaire has a sensitivity of 0.94, specificity of 0.79 (based on a clinical cut-off of AHI > 5), positive predictive value of 0.91 and negative predictive value of 0.86.

## Statistical Analysis

Frequency and measures of central tendency were used to describe the sample. In preliminary analyses, Pearson and Spearman correlations were used to explore relationships between variables of interest. A multivariate-adjusted logistic regression modeling was also used. Covariates entered in the model include age, sex, obesity (defined as BMI > 30 kg/m^2^), history of diabetes, dyslipidemia, hypertension and a measured waist circumference. Data were processed by an experienced Biostatistician using SPSS 20.0 (Chicago, SPSS Inc.).

## Results

The study revealed 93% of the sample were diagnosed with hypertension; 61%, diabetes; 72%, dyslipidemia; 90% were overweight/obese; 33% had a history of heart disease and 10% had a stroke. ARES data indicated that 48% were at high OSA risk. Logistic regression analysis, adjusting for age and gender, showed that patients at risk for high OSA had a nearly three-fold increase in odds of having a stroke (OR = 2.79, 95% CI: 1.64–4.73).

## Discussion

Results from this study revealed blacks with MetS had a 48 percent increased risk of OSA that accompanied a nearly three-fold increase in odds of having a stroke. In an observational cohort study, OSA was associated with stroke and death (hazard ratio, 1.97; 95 percent confidence interval, 1.12 to 3.48) [13,15–18]. Similar, findings were noted in the Vitoria Sleep Project, where it was determined that elderly patients with OSA were at a higher risk for stroke (hazard ratio [HR] =2.52, 95 % CI, 1.04–6.01, P = 0.04) [13]. OSA seemingly increases the intensity of “The Perfect Storm”, vascular comorbidities such as hypertension; atrial fibrillation and atherosclerosis have been proven to contribute the increase risk of stroke [13].

In patients with no medical history of OSA a 10%−15% nocturnal drop of blood pressure is usually experienced, however in patients with OSA the protective effect of nocturnal blood pressure dipping is lost [18]. As a result, during an apneic episode, hypoxia can induce vasodilatation or a reflex cerebral vasoconstriction, a mechanism that can lead to a stroke [13].

Although OSA plays a role in the progression of many diseases, it is uniquely important within the context of stroke. The prevalence of sleep apnea in stroke patients has been shown to be as high as 50–70% [16]. OSA is a predictor of both poor functional outcomes after a stroke and post-stroke mortality [19]. It is thought that poor cerebral blood flow from changes in blood pressure and oxygen saturation during apneic episodes affect the neurological recovery after cerebrovascular events. A greater than 50% reduction in middle cerebral artery blood flow velocity has been demonstrated by transcranial doppler studies during obstructive apneas and hypopneas [8]. A 2018 study showed that patients with more severe OSA as defined by the apnea-hypopnea index (AHI) demonstrated significantly increased levels of calcified carotid arterial plaques (CCAP) on panoramic imaging compared to patients with lower AHI scores, an important validated risk factor for future adverse cerebrovascular and cardiovascular events (OR = 1.035; CI, 1.008–1.062; P = .010). The same study also found almost a third of patients with OSA were found to have CCAP on panoramic imaging [20].

Studies have shown that blacks that are obese are more prone to develop OSA [13,15]. Additionally, obesity has been proven to be a top contributor to the development of MetS in blacks [21]. During an apneic hypopneic episode, sleep duration is altered, sleeping <6 hrs or >8 hrs; researchers have propositioned the idea of prolong sleep occurring as an attempt to compensate for fragmented sleep [13,16]. In addition, short and long sleep perpetuated by OSA has been linked to increase in stroke mortality and cardiovascular diseases [22,23]. Furthermore, blacks with MetS experience an increase in endothelial and microvascular damage [24].

Research has suggested the role of endothelial inflammation and damage to play a violent role regarding the pathophysiology of a stroke; resulting in thrombosis, due to increase D-dimers, fibrinogens, inflammatory markers and endothelial progenitor cells. Chu et al. 2008, set out to investigate the microvascular environment of endothelial progenitor cells (EPC) in 75 patients with acute stroke, 45 patients with chronic stroke and 40 patients deemed healthy with no history of stroke [24]. It was found that endothelial progenitor cells (EPC) was demonstrated in 71% of patients with acute stroke, 22% with chronic stroke and 10% of those healthy, concluding that endothelial cell injury and dysfunctions are predictors of increased risk of a vascular event [6,24]. Research has shown that both MetS and OSA can alter the microvascular environment [24–26]. A turbulent endothelial environment to increase the risk of stroke [27], furthermore the MetSO trial, the largest clinical trial to date of this kind, has provided unique insight of how blacks with MetS are 1) at increased risk for OSA and 2) the combination of both OSA and MetS may contribute significantly to increase risk of stroke in the black population.

## Conclusions

As research continues to increase patient and physician awareness of OSA, a deeper appreciation of the detrimental long-term consequences of diagnosing and treating OSA have increased in popularity. As it stands, the most predominate risk factor for MetS is obesity, which is also a dominate risk factor for OSA. Both MetS and OSA contribute to the destruction of the microvascular environment increasing the risk of stroke. Prevention and control is of the utmost importance, modification of risk factors such as obesity is an important public health concern.

## Figures and Tables

**Figure1:**
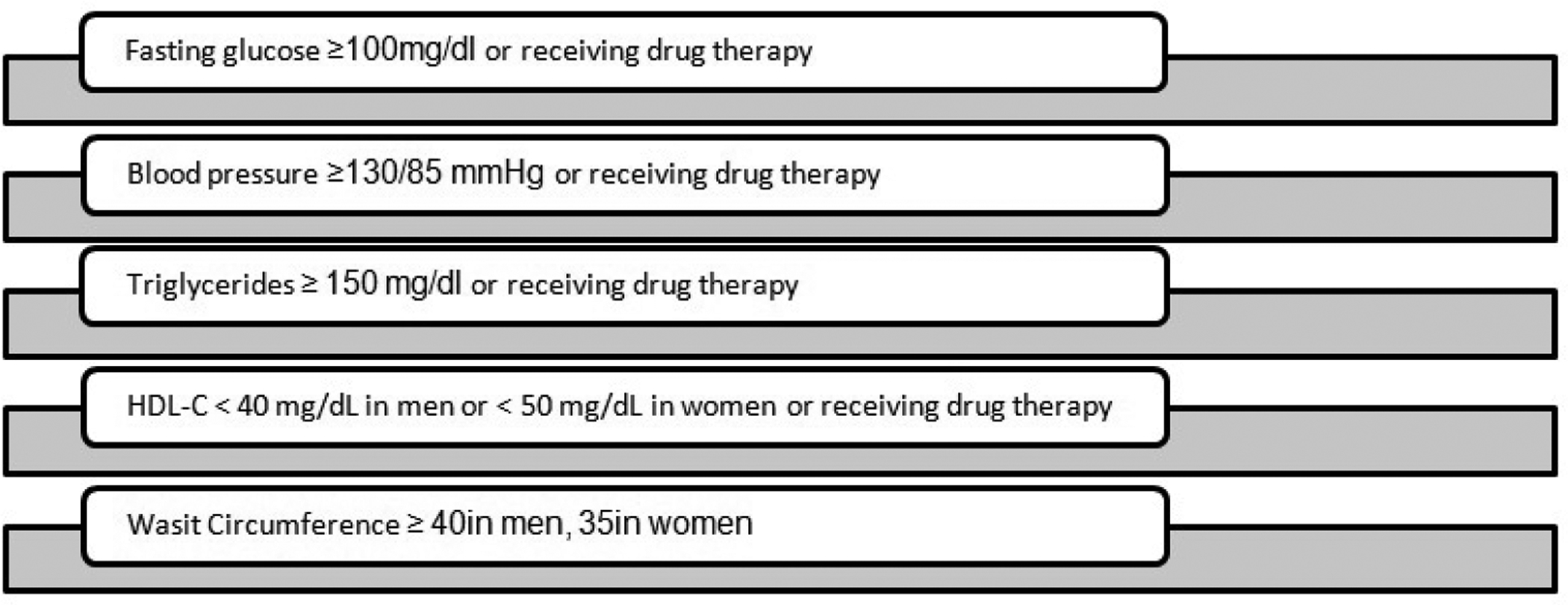
Criteria for metabolic syndrome: patients must have at least three of the following five conditions.

**Figure 2:**
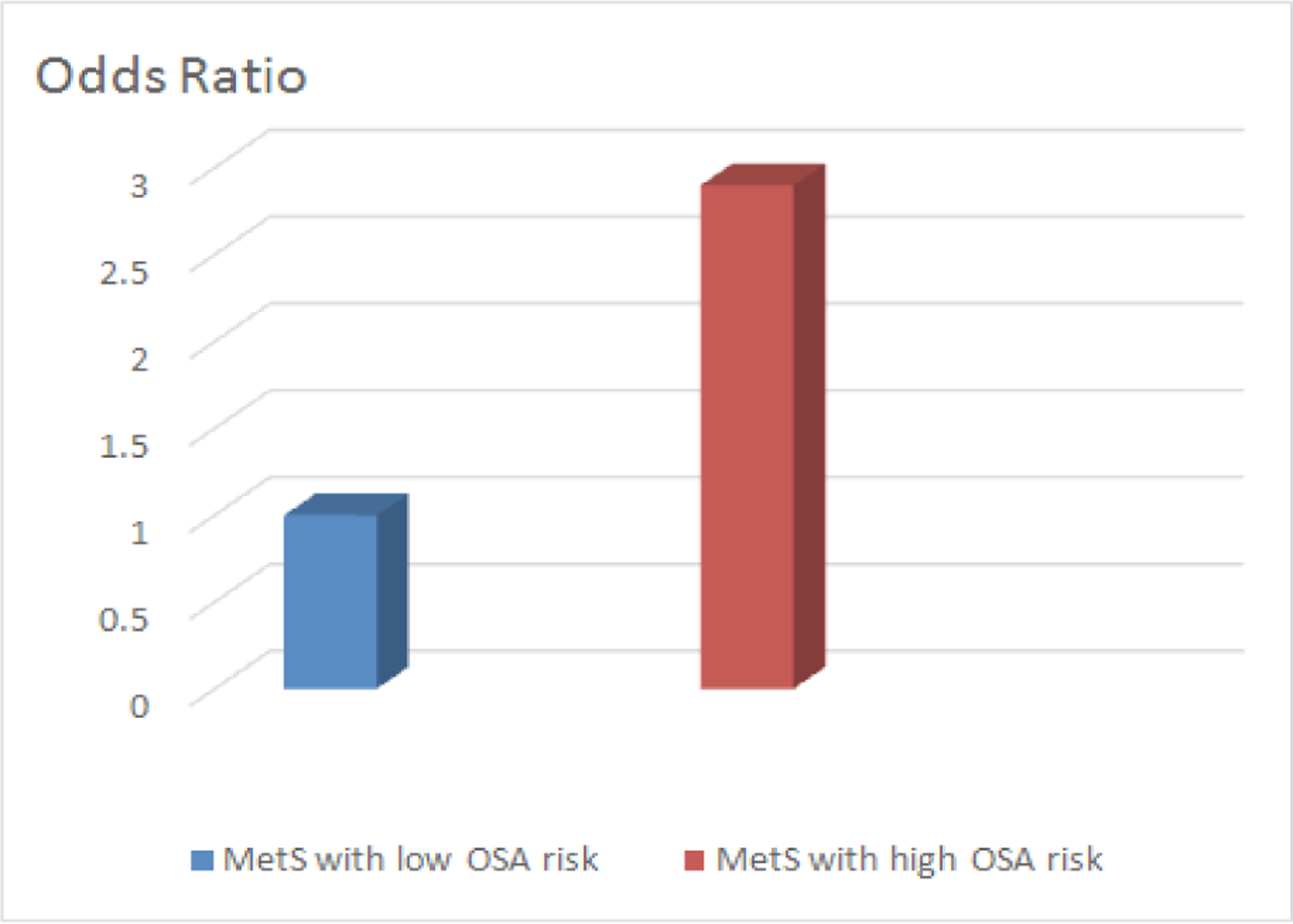
Participants in the Metabolic Syndrome Outcome study (MetSO) with high obstructive sleep apnea (OSA) risk (ARES score ≥ 6) demonstrated a near 3-fold increase risk of stoke when compared to participants with low (OSA) risk.

**Figure 3:**
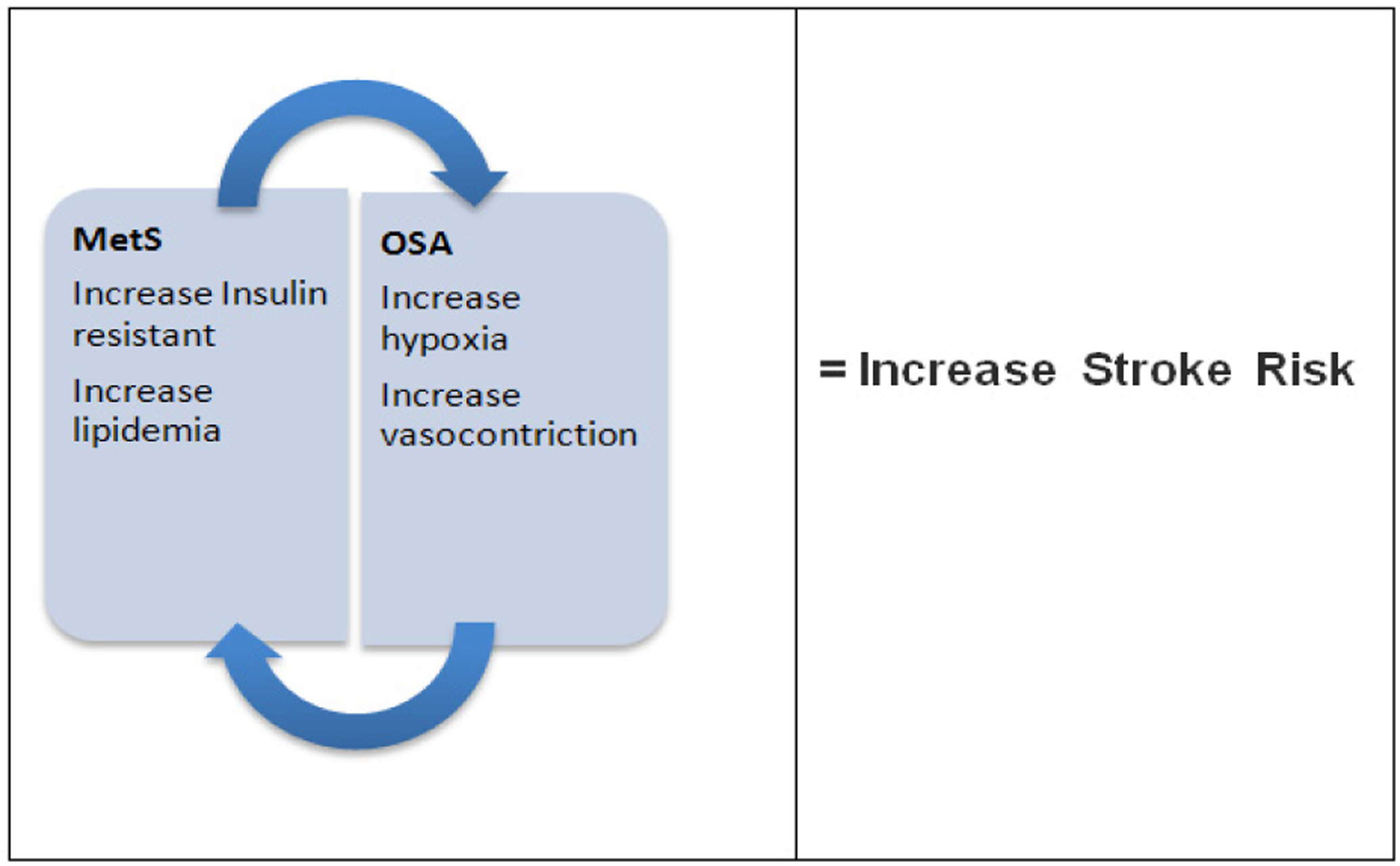
The plausible cyclic effect of metabolic syndrome and OSA resulting in increased risk of stroke.

**Table 1:** Metabolic characteristics of the study participants.

Variable	Mean	SD
Systolic BP	134.98	16.39
Diastolic BP	75.77	10.55
LDL Cholesterol	105.6	36.88
HDL Cholesterol	48.03	16.49
Triglycerides	134.98	73.24
Glucose	138.38	68.27
HbA1c	7.93	1.63

Note: BP= Blood Pressure; LDL= Low-density lipoprotein, HDL = High-density lipoprotein; HbA1c= glycated hemoglobin.

## References

[1] Quanhe Y, Xin T, Schieb L, Vaughan A, Gillespie C (2017) Vital Signs: Recent Trends in Stroke Death Rates-United States, 2000–2015. MMWR Morb Mortal Wkly Rep 66: 933–939. 28880858 10.15585/mmwr.mm6635e1 PMC5689041

[2] Hunt BR, Deot D, Whitman S (2014) Stroke mortality rates vary in local communities in a metropolitan area: racial and spatial disparities and correlates. Stroke 45: 2059–2065. 24876083 10.1161/STROKEAHA.114.005431

[3] Yan-fang S, Yu-ping W (2009) Sleep-disordered breathing: impact on functional outcome of ischemic stroke patients. Sleep med 10: 717–719. 19168390 10.1016/j.sleep.2008.08.006

[4] Labarthe D, Grover B, Galloway J, Gordon L, Moffatt S, (2014) The public health action plan to prevent heart disease and stroke: Ten-year update. Paper presented at: National Forum for Heart Disease and Stroke Prevention.

[5] Go AS, Mozaffarian D, Roger VL, Benjamin EJ, Berry JD, Executive summary: heart disease and stroke statistics-2014 update: a report from the American Heart Association. Circulation 129: 399–410. 24446411 10.1161/01.cir.0000442015.53336.12

[6] Das AM, Khan M (2012) Obstructive sleep apnea and stroke. Expert Rev Cardiovasc Ther 10: 525–535. 22458584 10.1586/erc.12.25

[7] Mehra R (2014) Sleep apnea ABCs: airway, breathing, circulation. Cleve Clin J Med 81: 479–489. 25085986 10.3949/ccjm.81gr.14002

[8] Davis AP, Billings ME, Longstreth W, Khot SP (2013) Early diagnosis and treatment of obstructive sleep apnea after stroke: Are we neglecting a modifiable stroke risk factor? Neurol Clin Pract 3: 192–201. 23914326 10.1212/CPJ.0b013e318296f274 PMC3721244

[9] Somers VK, White DP, Amin R, Abraham WT, Costa F, (2008) Sleep apnea and cardiovascular disease: An American heart association/American college of cardiology foundation scientific statement from the American heart association council for high blood pressure research professional education committee, council on clinical cardiology, stroke council, and council on cardiovascular nursing in collaboration with the national heart, lung, and blood institute national center on sleep disorders research (national institutes of health). Circulation 52: 686–717. 10.1016/j.jacc.2008.05.002 18702977

[10] Rogers AJ, Xia K, Soe K, Sexias A, Sogade F, (2017) Obstructive Sleep Apnea among Players in the National Football League: A Scoping Review. J Sleep Disord Ther 6: 278. 29984115 10.4172/2167-0277.1000278 PMC6035001

[11] Jean-Louis G, Zizi F, Clark LT, Brown CD, McFarlane SI, (2008) Obstructive sleep apnea and cardiovascular disease: role of the metabolic syndrome and its components. J Clin Sleep Med 4: 261–272. 18595441 PMC2546461

[12] Gaillard TR (2018) The metabolic syndrome and its components in African-American women: emerging trends and implications. Front Endocrinol 8: 383. 10.3389/fendo.2017.00383 PMC5786579 29403438

[13] Roth EJ, Lovell L (2014) Employment after stroke: report of a state of the science symposium. Top Stroke Rehabil 21: 75–86. 24722046 10.1310/tsr21S1-S75

[14] Alberti KG, Eckel RH, Grundy SM, Zimmet PZ, Cleeman JI, (2009) Harmonizing the metabolic syndrome: a joint interim statement of the international diabetes federation task force on epidemiology and prevention; national heart, lung, and blood institute; American heart association; world heart federation; international atherosclerosis society; and international association for the study of obesity. Circulation 120: 1640–1645. 19805654 10.1161/CIRCULATIONAHA.109.192644

[15] Barone DA, Krieger AC (2013) Stroke and obstructive sleep apnea: a review. Curr Atheroscler Rep 15: 334. 23666861 10.1007/s11883-013-0334-8

[16] Hermann DM, Bassetti CL (2003) Sleep-disordered breathing and stroke. Current opinion in neurology 16: 87–90. 12544862 10.1097/01.wco.0000053587.70044.be

[17] Lally F, Thakkar A, Roffe C (2011) Sleep apnoea and stroke. Somnologie-Schlafforschung und Schlafmedizin 15: 148.

[18] Lipford MC, Park JG, Ramar K (2014) Sleep-disordered breathing and stroke: therapeutic approaches. Curr Neurol Neurosci Rep 14: 431. 24395523 10.1007/s11910-013-0431-7

[19] Bassetti CL, Milanova M, Gugger M (2006) Sleep-disordered breathing and acute ischemic stroke: diagnosis, risk factors, treatment, evolution, and long-term clinical outcome. Stroke 37: 967–972. 16543515 10.1161/01.STR.0000208215.49243.c3

[20] Chang TI, Lee UK, Zeidler MR, Liu SY, Polanco JC, (2019) Severity of Obstructive Sleep Apnea Is Positively Associated With the Presence of Carotid Artery Atheromas. J Oral Maxillofac Surg 77: 93–99. 30213534 10.1016/j.joms.2018.08.004

[21] Fülöp T, Hickson DA, Wyatt SB, Bhagat R, Rack M, (2012) Sleep-disordered breathing symptoms among African-Americans in the Jackson Heart Study. Sleep Med 13: 1039–1049. 22841028 10.1016/j.sleep.2012.06.005 PMC3427405

[22] Youngstedt SD, Kripke DF (2004) Long sleep and mortality: rationale for sleep restriction. Sleep Med Rev 8: 159–174. 15144959 10.1016/j.smrv.2003.10.002

[23] Munoz R, Duran-Cantolla J, Martínez-Vila E, Gallego J, Rubio R, Severe sleep apnea and risk of ischemic stroke in the elderly. Stroke 37: 2317–2321. 10.1161/01.STR.0000236560.15735.0f 16888274

[24] Chu K, Jung KH, Lee ST, Park HK, Sinn DI, (2008) Circulating endothelial progenitor cells as a new marker of endothelial dysfunction or repair in acute stroke. Stroke 39: 1441–1447. 18356550 10.1161/STROKEAHA.107.499236

[25] Grundy SM, Brewer HB Jr, Cleeman JI, Smith SC Jr, Lenfant C, (2004) Definition of metabolic syndrome: report of the National Heart, Lung, and Blood Institute/American Heart Association conference on scientific issues related to definition. Arterioscler Thromb Vasc Biol 109: 433–438. 10.1161/01.ATV.0000111245.75752.C6 14766739

[26] Kato M, Roberts-Thomson P, Phillips BG, Haynes WG, Winnicki M, (2000) Impairment of endothelium-dependent vasodilation of resistance vessels in patients with obstructive sleep apnea. Circulation 102: 2607–2610. 11085964 10.1161/01.cir.102.21.2607

[27] Netzer N, Werner P, Jochums I, Lehmann M, Strohl KP, (1998) Blood flow of the middle cerebral artery with sleep-disordered breathing: correlation with obstructive hypopneas. Stroke 29: 87–93. 9445334 10.1161/01.str.29.1.87

[28] Khot SP, Davis AP, Crane DA, Tanzi PM, Lue DL, (2016) Effect of continuous positive airway pressure on stroke rehabilitation: a pilot randomized sham-controlled trial. J Clin Sleep Med 12: 1019–1026. 27092703 10.5664/jcsm.5940 PMC4918984

[29] Gupta A, Shukla G, Afsar M, Poornima S, Pandey RM, (2018) Role of positive airway pressure therapy for obstructive sleep apnea in patients with stroke: a randomized controlled trial. Journal of Clinical Sleep Medicine 14: 511–521. 29609704 10.5664/jcsm.7034 PMC5886428

